# Disinfectants, and How to Use Them

**Published:** 1879-10

**Authors:** 


### Miscellaneous.

### DISINFECTANTS, AND HOW TO USE THEM.

The National Board of Health, consisting of a number of our leading physicians and chemical experts, of which Prof. C. F. Chandler is chairman, have issued the following instructions for disinfection, intended especially for the guidance of physicians and nurses in the yellow fever districts, but which are equally applicable in other classes of contagious diseases. In submitting this report, the chairman says:

It has been the aim of the committee to prepare concise directions for disinfection, so simple and clear that they may be easily followed by any person of intelligence.

In the selection of disinfecting agents the aim has been: 1st, to secure agents which can be relied upon to accomplish the work; 2d, which can be procured in a state of comparative purity in every village in the United States; 3d, so cheap that they can be used in adequate quantities.

It is extremely important that the people should be instructed with regard to disinfection. They must be taught that no reliance can be placed on disinfectants, simply because they smell of chlorine or carbolic acid, or possess the color of permanganate, and that, in general, proprietary disinfectants with high-sounding names are practically worthless, as they either have no value whatever, or, if of value, cost many times as much as they are worth, and cannot be used in sufficient quantity.

### EXPLANATIONS.

Disinfection is the destruction of the poisons of infectious and contagious diseases.

Deodorizers, or substances which destroy smells, are not necessarily disinfectants, and disinfectants do not necessarily have an odor.

Disinfection can not compensate for want of cleanliness or of ventilation.

### I.—DISINFECTANTS TO BE EMPLOYED.

1. Roll sulphur (brimstone) for fumigation.

2. Sulphate of iron (copperas) dissolved in water in the proportion of one and a half pounds to the gallon; for soil, sewers, etc.

3. Sulphate of zinc and common salt, dissolved together in water in the proportions of four ounces sulphate and two ounces salt to the gallon; for clothing, bed linen, etc.

NOTE.—Carbolic acid is not included in the above list for the following reasons: It is very difficult to determine the quality of the commercial article, and the purchaser can never be certain of securing it of proper strength; it is expensive, when of good quality, and experience has shown that it must be employed in comparatively large quantities to be of any use; it is liable by its strong odor to give a false sense of security.

### II.—HOW TO USE DISINFECTANTS.

1. In the Sick-Room.—The most available agents are fresh air and cleanliness. The clothing, towels, bed linen, etc., should at once, on removal from the patient, be placed in a pail or tub of the zinc solution, boiling hot if possible, before removal from the room.

All discharges should either be received in vessels containing copperas solution, or, when this is impracticable should be immediately covered with copperas solution. All vessels used about the patient shou'd be cleansed with the same solution.

Unnecessary furniture—especially that which is stuffed—carpets, and hangings, when possible, should be removed from the room at the outset; otherwise, they should remain for subsequent fumigation and treatment.

2. Fumigation with sulphur is the only practicable method for disinfecting the house. For this purpose the rooms to be disinfected must be vacated. Heavy clothing, blankets, bedding, and other articles which can not be treated with zinc solution, should be opened and exposed during fumigation, as directed below. Close the rooms as tightly as possible, place the sulphur in iron pans supported upon bricks, set it on fire by hot coals, or with the aid of a spoonful of alcohol, and allow the room to remain closed for twenty-four hours. For a room about ten feet square, at least two pounds of sulphur should be used; for larger rooms, proportionally increased quantities.

3. Premises.—Cellars, yards, stables, gutters, privies, cesspools, water closets, drains, sewers, etc., should be frequently and liberally treated with copperas solution. The copperas solution is easily prepared by hanging a basket containing about sixty pounds of copperas in a barrel of water.

4. Body and bed clothing, etc.—It is best to burn all articles which have been in contact with persons sick with contagious or infectuous diseases. Articles too valuable to be destroyed, should be treated as follows:

a. Cotton, linen, flannels, blankets, etc., should be treated with the boiling hot zinc solution, introducing piece by piece, securing thorough wetting, and boiling for at least half an hour.

b. Heavy woolen clothing, silks, furs, stuffed bed covers, beds, and other articles which can not be treated with the zinc solution, should be hung in the room during fumigation, pockets being turned inside out, and the whole garment thoroughly exposed. Afterward they should be hung in the open air, beaten, and shaken. Pillows, beds, stuffed mattresses, upholstered furniture, etc., should be cut open, the contents spread out and thoroughly fumigated. Carpets are best fumigated on the floor, but should afterward be removed to the open air and thoroughly beaten.

5. The corpses should be thoroughly washed with a zinc solution of double strength, then wrapped in a sheet wet with the zinc solution, and buried at once. Metallic, metal-lined, or air-tight coffins should be used when possible, certainly when the body is to be transported for any considerable distance.

